# Deep brain stimulation of the ventral striatum/anterior limb of the internal capsule in thalamic pain syndrome: study protocol for a pilot randomized controlled trial

**DOI:** 10.1186/1745-6215-14-241

**Published:** 2013-07-31

**Authors:** Ela B Plow, Donald A Malone, Andre Machado

**Affiliations:** 1 Department of Biomedical Engineering, Department of Physical Medicine and Rehabilitation and Center for Neurological Restoration, Cleveland Clinic, 9500 Euclid Avenue, ND20, Cleveland, OH 44195, USA; 2 Department of Psychiatry & Psychology and Center for Behavioral Health/P57, Neurological Institute, Cleveland Clinic, 9500 Euclid Avenue, Cleveland, OH 44195, USA; 3 Center for Neurological Restoration, Department of Neurological Surgery/S31, Neurological Institute, Cleveland Clinic, 9500 Euclid Avenue, Cleveland, OH 44195, USA

**Keywords:** Chronic pain, Neuropathic pain, Stroke, Thalamic pain syndrome, Deep brain stimulation (DBS), Ventral striatum, Anterior limb of internal capsule, Post-stroke pain, Central pain, Brain stimulation

## Abstract

**Background:**

Chronic neuropathic pain in thalamic pain syndrome remains intractable. Its poor response is ascribed to destruction of the integrated neuromatrix in experience of pain. Deep brain stimulation is a promising technique to modulate activity of implicated structures. However, traditional approaches targeting sensori-motor substrates have failed to affect disability. The offending lesion in thalamic pain syndrome that almost invariably destroys sensory pain pathways may render these classical approaches ineffective. Instead, we hypothesize that targeting structures representing emotion and affective behavior-ventral striatum/anterior limb of the internal capsule, may alleviate disability.

**Methods/design:**

We present the design of our phase I randomized, double-blinded, sham-controlled, crossover trial that examines safety, feasibility and efficacy of our proposed approach. In our ongoing trial, we intend to enroll ten patients with thalamic pain syndrome. Following implantation, patients are randomized to receive active deep brain stimulation to the ventral striatum/anterior limb of the internal capsule or sham for 3 months, after which they are crossed over. The primary endpoint is Pain Disability Index. Other outcomes include visual analog scale, depression and anxiety inventories, quality of life, and functional neuroimaging.

**Discussion:**

Designing trials of deep brain stimulation for pain is challenging owing to the ethical-scientific dilemma of introducing a control arm, complicated blinding, heterogeneous etiologies, patient expectations, and inadequate assessment of disability. The quality of evidence in the field is classified as level III (poor) because it mainly includes a multitude of uncontrolled case series reporting variable outcomes, with little regard for the placebo effect related to implantation. Without valid data on efficacy, use of deep brain stimulation for pain remains “off label”. We present our trial design to discuss feasibility of conducting sham-controlled phase I studies that may represent significant refinement for the field. Double-blinding would reduce influence of patient expectations and therapeutic confusion amongst investigators. With a cross-over approach, the dilemma regarding including a control group can be mitigated. Use of homogeneous etiology, measurement of disability, depression and quality of life, besides pain perception, all represent strategies to evaluate efficacy rigorously. Functional imaging would serve to define mechanisms underlying observed effects and may help optimize future targeting.

**Trial registration:**

Clinicaltrials.gov NCT01072656

## Background

### Thalamic pain syndrome: refractoriness to traditional management

Chronic neuropathic pain, defined as ‘pain initiated or caused by a primary lesion or dysfunction in the nervous system’ [1], is one of the most prevalent disorders, affecting about 8% of adults [2]. Although it can be associated with peripheral or central pathology, pain of central origin, such as that following stroke, is more resistant to treatment [3-5]. In fact, pain associated with lesions of somatosensory thalamic nuclei or somatosensory thalamo-cortical projections, following subcortical stroke, is particularly intractable and extraordinarily frustrating for patients and physicians. Also referred to as thalamic pain syndrome (TPS) or Dejerine-Roussy syndrome, the disorder is characterized by unrelenting pain, associated paradoxically with numbness (anesthesia dolorosa) on the affected side. Its prevalence is estimated to be 8% across all stroke patients but can be as high as 18% in those with sensory deficits [6].

Poor responsiveness in TPS is attributed to disruption of the pain neuromatrix [7]. The neuromatrix is an integrated framework of somatosensory, limbic and cognitive networks in the brain interacting with thalamo-cortical pathways in the experience of pain. Since mechanisms of pain in TPS span across such widespread cerebral structures within the neuromatrix, a promising technique involves modulating activity of these structures with invasive deep brain stimulation (DBS) [8].

Almost four decades ago [9] DBS was introduced to target structures involved in pain transmission and opioid-mediated analgesia, such as the periventricular and periaqueductal gray matter. Fifteen years later, results continued to be disappointing in TPS [4] even though response across other etiologies was staggering. Emphasis was then shifted to target the sensory thalamic nodes based on the premise that stimulating the thalamus would inhibit hyperactivity within pain-transmitting neuronal fibers associated with unrelenting pain in TPS [10]. DBS of the ventral posterior nuclei of the thalamus, however, generated inconsistent benefit [4,5]. More recently, motor cortex stimulation (MCS) was introduced as a less invasive alternative, based on the hypothesis that motor cortical influence via cortico-thalamic pathways would reduce thalamic hyperactivity in pain [11]. Tsubokawa *et al*. initially demonstrated positive results across several central pain syndromes [12], but promise for patients with TPS was subsequently irreproducible [13-16]. Thus, it is critical to re-evaluate substrates that have been targeted with DBS, to understand and resolve reasons for their failure in TPS over the last 40 years.

### Importance of identifying novel nodes for targeting with DBS in TPS

We suggest there may be several reasons for failure of traditional DBS that targets sensorimotor substrates in management of TPS. First, the offending lesion in TPS damages sensorimotor targets such as the ventral-posteromedial and ventral-posterolateral targets that are critical to conduct the effects of contemporary DBS [11,17]. Second, since pain experience and disability is an integrative phenomenon of nociception influenced by affect and cognition [7], substrates that are key for regulation of emotion and behavior, such as ventral striatum (VS) and anterior limb of the internal capsule (ALIC), could be more effective in influencing the experience of disability. Patients with TPS experience severe allodynia and suffer from learned behaviors of anticipating pain, which exacerbates disability. DBS that modulates VS/ALIC may modify learned affective behavioral responses [18] as in obsessive compulsive disorder [19] and depression [20] and, thus, would potentially be more effective in mitigating pain-related disability.

### Challenges in designing a clinical trial of DBS in treatment of TPS

Our group has recently suggested departing from the traditional approach of targeting DBS to sensorimotor substrates and instead provided rationale for a novel DBS target involving VS/ALIC in the management of chronic neuropathic pain in TPS [21]. Examining efficacy of a novel node of DBS in a population with refractory chronic neuropathic pain, however, is challenging because the paradox of experience-subjectivity of pain affects assessment of outcomes, while patient expectations from the investigational procedures as well as surgical and neuro-stimulation interventions may generate several confounds. The quality of contemporary evidence regarding effectiveness of DBS in pain is currently classified as level III because studies thus far, have enrolled heterogenous etiologies, incorporated inadequate experimental blinding and controlled poorly for confounds of placebo. In addition, little regard has been given to the risk for therapeutic confusion in the investigative team about benefits of treatment, or to key outcome measures that evaluate the experience/disability of pain or its effect on quality of life, rather than severity on a numerical scale of pain perception [8,22].

### Objectives and hypothesis

Here, we present the protocol design of our current pilot clinical trial where we examine: 1) safety and feasibility of delivering DBS to VS/ALIC in a double blinded, randomized, sham-controlled, crossover clinical trial in patients with TPS; 2) efficacy of the intervention with respect to pain-related disability, quality of life, neuropsychological and psychiatric outcomes and individual ratings of perception of pain; and 3) reorganization within affective networks of the pain neuromatrix with DBS delivered to VS/ALIC, which will be studied using functional magnetic resonance imaging (fMRI) and magneto-encephalography (MEG).

We hypothesize that DBS targeting VS/ALIC will modulate the affective component of TPS and consequently, improve pain-related disability.

## Methods

### Study design

A pilot, randomized, sham-controlled, double-blinded, two-group crossover clinical trial design is being employed to evaluate the safety and effectiveness of DBS targeting VS/ALIC in TPS. Patients with TPS are being enrolled, and then randomized to active DBS or sham following implantation, after which they are crossed over. The study has been approved by the Food and Drug Administration with an Investigational Device Exemption as a phase I clinical trial, and is registered (http://clinicaltrials.gov/ct2/show/NCT01072656) as a clinical trial. The Institutional Review Board of the Cleveland Clinic has approved the study. Informed consent is obtained from all interested participants in accordance with policies of the Board.

### Patient population and recruitment

We are actively enrolling for this study. We intend to enroll a total of 10 patients with TPS, such as that following stroke, surgical injury or focal traumatic injury, proven refractory to conventional pain management. The selection criteria are developed to enroll patients with intractable pain contralateral to a brain lesion, without severe psychiatric or cognitive comorbidities, suicide ideation, major uncontrolled medical problems or previous ablative surgery for TPS. Patients who have had motor cortex stimulation (MCS) but not DBS are also candidates, provided that the MCS hardware has been removed. Inclusion and exclusion criteria are discussed in detail below.

#### Inclusion criteria

•Age > 21 years

•Diagnosis of TPS >6 months

•MRI-confirmed lesion affecting the posterior thalamus or dorsal or ventral vicinity of thalamus or brain stem

•Patients with lesions involving the semi-oval white matter in topography consistent with sensory thalamo-cortical connections and presenting with clinical syndrome consistent with TPS

•Chief complaints of intractable hemibody pain that has remained at an average score >5 on the visual analog scale (VAS, scores ranging between 0 and 10) for 30 days, pinprick hypoesthesia over the affected hemibody (anesthesia dolorosa), severe allodynia, significant pain-related disability (Pain Disability Index or PDI score >30), failure to respond to at least one medication within each class of drugs prescribed commonly for central pain syndrome, including antidepressants, anti-seizure medications and oral narcotics.

#### Exclusion criteria

•Issues with psychiatric health, including inability to cope with implantable hardware, severe uncontrolled depression, bipolar disorder, severe obsessive compulsive disorder, suicide ideation within 12 months prior to enrollment or communication of a plan should the investigational treatment fail or imminent suicide risk (measured based on the Montgomery-Asburg Depression Rating Scale (MADRS) and the Columbia-Suicide Severity Rating Scale (C-SSRS) and the Beck Depression Inventory (BDI)

•Unstable physical health, other neurological lesions or MRI-based incidental findings unrelated to the offending lesion, women of childbearing age who are pregnant at the time of enrollment or are not using adequate contraception

•Co-morbidities including ongoing anticoagulation/anti-aggregation therapy, uncontrolled hypertension or major organ system failure

•Prior or concurrent therapies related to TPS, including previous ablative intracranial surgery for management of TPS, previous DBS therapy or simultaneous enrollment in another clinical trial for TPS

•Issues with cognitive health or ability to communicate, such as inability to provide informed consent, severe aphasia or inability to comply with the logistical requirements of the research study

Patients are being recruited from the Cleveland Clinic Neurological Institute Centers and from the Cleveland Clinic Pain Management Center. Other institutions in Northeastern Ohio and neighboring states have also been contacted about the study. These centers have access to the objectives of the study, intended population and study criteria, but patients are only enrolled and treated at the Cleveland Clinic.

### Study protocol: phases of the study

#### Phase I: screening and enrollment

Screening involves standard review of history, physical and neurological examination, review of MRI within the past year, pain questionnaires, psychiatric evaluation, pain psychological evaluation and laboratory tests. Patients meeting selection criteria are enrolled.

#### Phase II: pre-operative baseline evaluation

**Figure 1:**
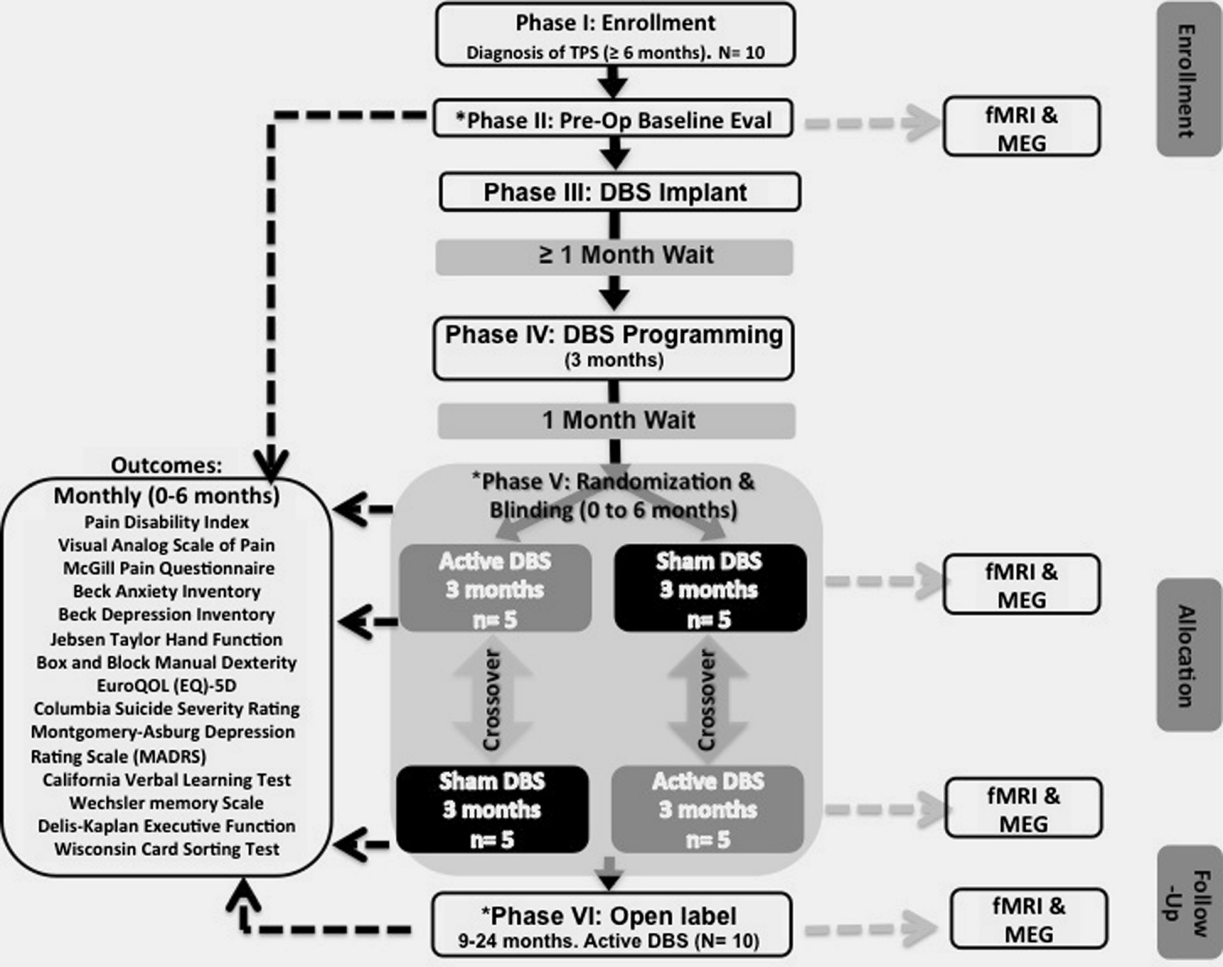
**Study design.** Flowchart of the study design based on specified CONSORT clinical trial guidelines [23]. Categories defined in CONSORT guidelines are marked on the right margin of the figure. ^*^Time points when neurological, neuropsychological pain or neuropsychological cognitive evaluations (Table 1) will be conducted. During phase V, fMRI and MEG evaluations will be conducted at 2 and 5 months after randomized intervention allocation. During the open-label phase, follow-up assessments are conducted at month 9, 12, 18 and 24 for outcomes listed above and at month 12 and 24 for fMRI and MEG. TPS, thalamic pain syndrome; DBS, deep brain stimulation; fMRI: functional magnetic resonance imaging; MEG: magneto-encephalography; EuroQOL (EQ-5D), Euro Quality of Life scale.

Phase II, or pre-operative baseline evaluation (Figure 1), involves collection of several clinical outcome measures, including extensive neurological, neuropsychological pain and cognitive and psychiatric assessments (Figure 1, Table 1). These assessments are performed before implantation, and repeated during the randomized phase, and again during the open-label follow-up phase. These evaluations are conducted by the same specialized team that routinely assesses neurologic, neuropsychological, cognitive and neuropsychiatric function in our clinical program and in our research studies in DBS. Patients also undergo functional neuroimaging (Figure 1).

**Table 1:** **Neurological**, **neuropsychological and psychiatric examination**

**Neurological examination**:	Sensory and motor systems
**Neuropsychological Pain Evaluation**:	Coping skills and personality assessment
**Neuropsychological Cognitive Assessment and Psychiatric Evaluation**:	a) California Verbal Learning Test
	b) Wechsler memory Scale
	c) Delis-Kaplan Executive Function System
	d) Wisconsin Card Sorting Test
	e) Beck Depression Inventory
	f) Beck Anxiety Inventory
	g) Columbia Suicide Severity Rating Scale (CSSRS)
	h) Montgomery-Asburg Depression Rating Scale (MADRS)
	i) Twenty Item Positive and Negative Affect Schedule (PANAS) and NEO Five Factor Inventory (NEO_FFI)

Neurological, neuropsychological pain and neuropsychological cognitive and psychiatric examinations; marked by an asterisk in Figure 1, these examinations will be conducted at pre-operative baseline, at the time of randomization and at month 12 and 24 following randomization (well into phase VI follow-up testing).

#### Phase III DBS implantation

All enrolled patients receive DBS implantation bilaterally in the VS/ALIC target. DBS lead implantation is completed in the same fashion as previously described by our group, but for the VS/ALIC target [18,24]. The VS/ALIC is targeted approximately at its junction with the anterior commissure and the electrodes are advanced further ventrally along the trajectory towards the VS. Once inserted, intraoperative testing with macrostimulation through the DBS lead is performed. Based on prior experience [20], we have noted that the most ventral electrode contacts elicit acute changes in mood or anxiety. At this stage, it is unknown if stimulation also produces changes in pain perception. If no effects are seen, the implanting team may consider revising the location of the lead(s). Once a location is identified, DBS leads are anchored to the skull and surgical sites are closed. The implantable pulse generator (IPG) is positioned in the infraclavicular area and connected to DBS leads under general anesthesia. Since the surgical intervention and general anesthesia may be a risk for cognitive decline, we serially assess cognitive function to note whether any patients experience negative effects on cognition related to our interventions (see Table 1).

#### Phase IV: DBS programming and titration

DBS programming is initiated at least a month after DBS surgery to allow for resolution of microlesional effects. In acute programming, patients are asked to report changes in mood, anxiety, pain or suffering with activation of one or more electrode contacts. If the affective reporting of pain is not influenced, then settings affecting mood and anxiety can be chosen for chronic stimulation. All stimulation settings are planned below a charge density of 30 μC/cm^2^/phase, using the formula:

$$\begin{array}{c} \text{Charge}\;\text{density}\;\left({\mathrm{\mu C}/\mathrm{cm}}^{2}/\text{phase}\right)\\ =\frac{\text{Voltage}\;\left(V\right)*\text{Pulse}\;\text{width}\;\left(\mathrm{\mu s}\right)}{\text{Lead}\;\text{surface}\;\text{area}\;\left({\mathrm{cm}}^{2}\right)*\text{Impedance}\;\left(Ω\right)}\;\mathrm{Eq}.\;1 \end{array}$$

#### Phase V: randomization and intervention

Once optimal settings are determined, stimulation is turned off for about a month in order to minimize the effects of experience and memory of stimulation. Patients are then randomized to either the DBS group that receives active stimulation programmed to the settings found to be optimal during titration in phase IV, or to the sham group that receives 0 V (no electrical current) stimulation. As a technical detail, for minimizing risk to blinding procedures, the pulse generator is always kept on during all phases so that if any patients have tests that could indicate the status of the generator, it will always appear to be turned on Patients are randomized in a 1:1 ratio to one of the two groups.

#### Phase VI: open-label phase

Upon completion of the third month post-crossover, all patients are reprogrammed to the optimized stimulation settings, as deemed necessary by the investigators at that time to maximize clinical benefits. There is no sham stimulation after this point. The goal is to reduce pain-related disability. Follow-up assessment of outcomes is conducted at month 9, 12, 18 and 24.

### Endpoints of safety

Risks can be related to the procedures for implantation of DBS hardware, or the stimulation of the target area. Additional risks could be related to conducting MRI with implanted DBS. Events or instances that can present as risks are noted in detail in Table 2. Clinical experience of the research team, pre-operative image guidance, prophylactic antibiotics, and preparatory laboratory work is critical in mitigating risks related to DBS and implantation. Risks of stimulation are mitigated by choosing settings below the threshold for intolerable side effects [19]. Risk for suicidal ideation is monitored throughout the trial using C-SSRS. The protocol is outlined such that if serious signs are detected, psychiatric assessment will promptly follow. If the risk remains high for more than 28 days or if two or more events of imminent risk are recorded, then the individual will be removed from the blinded phase of the study.

**Table 2:** Overall risks of the study across various categories

**Category of Risks**	**Specific Risks**
**Risks of DBS surgery and Implantation of Pulse Generator**	Intracerebral hemorrhage; infection abscess; meningitis; misplacement of leads requiring revisions; post-operative displacement of leads; reaction to lead materials; hypodensity with mass effect around lead; intracranial extra-axial hemorrhage; stroke; pain at site of surgery; migration of leads; erosion of skin over hardware; hardware problems, such as abnormal impedances; hydrocephalus; seizures/epilepsy; cerebrospinal fluid leak; seroma; collection formation; pneumothorax/hemothorax/pleural effusions due to IPG; injury along the tunneling trajectory in the region of head, neck or chest; risks related to anesthesia (severe injury, cognitive decline, and death)
**Risks Pertaining to Hardware Maintenance**	Migration; infection; hardware malfunction/break; insulation problems; leaks of electricity; reaction to implants; erosion; incompatibility with MRIs; incompatibility with surgical equipment, medical devices or diagnostic devices
**Risks due to stimulation and conduct of the experiment**	Stroke/brain hemorrhage; dizziness; dyspraxia, dystonia, facial and limb muscle weakness, abnormal voluntary movements; numbness; attention, memory or language processing difficulties; suicide ideation and imminent risk of suicide (based on MADRS’ item 10, CSSRS’ item 5, BDI’s item 9 and behavioral interview); hypomania or motor contractions; any other unanticipated neurological deficits; worsening of chronic pain condition; behavioral or cognitive changes; rebound of symptoms when stimulation is stopped; visual field defects or eye movement disorders; headache; abnormal thinking; behavioral or psychiatric problems; changes in sexual function; disorders of consciousness; cardiovascular or respiratory events; electromagnetic interference; MRI may damage implantable devices
**Risks Involved with Imaging**	MRI may cause lesion to region targeted by DBS; fatigue and discomfort associated with nociceptive stimuli

IPG, implantable pulse generator; MADRS, Montgomery-Asburg Depression Rating Scale; C-SSRS, Columbia Suicide Severity Rating Scale; BDI, Beck Depression Inventory; MRI, Magnetic Resonance Imaging.

### Feasibility of blinding

Our current trial is a double-blinded study where neither patients nor investigators performing outcome assessments know whether DBS or sham is delivered. Maintaining a blinded study is important because of the subjective nature of reports of pain and disability. Loss of blinding may occur due to the magnitude of the therapeutic effect or any other subjective perception that indicates that active DBS or sham is delivered. Safeguards have been incorporated to prevent loss of experimental blinding; for instance, stimulation is turned to 0 V after the programming and titration in phase IV to minimize the chance of memory of stimulation affecting the patient’s perception of receiving active DBS or sham in blinded phase V. In addition, patients and blinded investigators are asked separately via questionnaire, what they believe is the status of the stimulation (active or sham). At the end of the study, we will evaluate how effective the blinding strategy was in order to guide us forward.

### Outcomes of efficacy

Outcomes of pain, pain-related suffering and disability, depression and anxiety, functional status, motor abilities, and suicidal ideation are collected at several time points (Figure 1).

#### Primary endpoint

Instead of using the historically accepted criterion of 50% analgesia (using the VAS), we instead chose a 40% effect on the PDI as our primary endpoint. The PDI measures disability in physical and psychosocial categories spanning across domains such as family, sexual, social, recreational, self-care, occupational and life-support domains [25]. Its strong relationship with quality of life outcomes makes it equivalent and potentially superior to existing functional-status questionnaires used in pain research, such as the VAS [26].

#### Secondary endpoints

Secondary endpoints include: 1) 40% improvement in PDI in 50% of patients at 2-year follow up; 2) affirmative response to the prospect of receiving the treatment again if the same outcome were to be achieved; and 3) 50% reduction in VAS at 2-year follow up.

Other outcomes include neuropsychological pain, and cognitive and psychiatric assessments (Figure 1, Table 1) as follows:

•The McGill Pain Questionnaire consists of descriptors of pain, including sensory, affective, evaluative and miscellaneous categories [27]

•The Beck Depression and Anxiety Inventories are self-report measures to document the level of depression [28] and anxiety and have been used in DBS studies of chronic neuropathic pain [5]

•Tests of manual dexterity: the box and block test specifically measures the grasp and release function of the paretic hand.

•EuroQOL (EQ-5D VAS) is a measure of health-related quality of life that has been employed in previous DBS studies in neuropathic pain [29]

•C-SSRS [30] provides valid, sensitive and specific assessment of suicidal ideation and behavior

•MADRS is a valid, acceptable, reliable and sensitive patient-reported outcome assessing severity of depression [31]

•Neuropsychological tests of cognitive function will include the California Verbal Learning Test, the Wechsler memory Scale, the Delis-Kaplan Executive Function System and the Wisconsin Card Sorting Test

In functional neuroimaging, fMRI and MEG are utilized to help define affective-cognitive networks associated with allodynia and anticipation of pain in chronic TPS, and their potential modulation with DBS of the VS/ALIC; fMRI has been performed safely in patients with DBS implants in our previous studies [32]. Our group has demonstrated that DBS delivered specifically to the VS/ALIC is associated with cortical-subcortical fMRI activation that represents affective networks in the brain [32]. MEG use with implanted DBS has also been shown to be safe and feasible [33,34]. We chose to utilize both fMRI and MEG to reveal substrates of affective dimension of pain because their unique strengths are complementary. Whereas fMRI is associated with superior spatial resolution, MEG possesses very high temporal resolution [35].

Both MEG and fMRI are exploratory in this study. With fMRI, one of our goals is to evaluate resting state fMRI, comparing networks when DBS is turned on versus when it is turned off. Patients will undergo resting-state fMRI at pre-operative baseline, during the randomized blinded phase and during follow up. Thus, by comparing the DBS-on and DBS-off conditions, we will be able to assess the effects of active versus sham DBS without confounds related to placebo or study-related effects. The next goal is to correlate changes in the neural networks related to the placebo effect; we intend to do so by comparing effects of DBS-off stimulation between active, sham and pre-operative baseline phases. With MEG, we aim to harness its high temporal resolution to assess possible effects of DBS on pain anticipation. The primary hypothesis is that DBS will have a significant effect upon the affective sphere of chronic pain. Pain anticipation is one of the conditions leading to pain habituation and chronic pain. Thus, we theorize that improvements in the affective sphere will also be correlated to how cortical networks in patients with TPS modulate in pain anticipation. In order to test this concept, we have created a visual paradigm of pain anticipation that consists of a simple, three-second countdown. The type of visual paradigm encodes whether or not a painful stimulus would follow the countdown. One of the limitations related to this paradigm is that we do not have a full characterization of how healthy individuals respond to pain anticipation in this task. This can limit our understanding of how processing of pain anticipation is abnormal in individuals with TPS, and could as well limit the interpretation of effects of active DBS. In order to address these limitations, we are also evaluating a cohort of 10 healthy subjects to allow comparisons.

### Statistical and data analysis plan

#### Safety and feasibility

The goal of this study is to evaluate the safety and feasibility of delivering DBS to the VS/ALIC in patients with TPS in a double-blinded, randomized, sham-controlled, crossover clinical trial. A Data Safety and Monitoring Board (DSMB) has been established as an advisory group to evaluate adverse events and judge overall integrity and conduct of the study. DSMB review at the outset, half-way through the enrollment process and at the end, will record numbers of participants enrolled, participants lost to follow up, deviations from protocol, threats to blinding and adverse events, following which, decisions about continuation or recommendations for changing or ending the study could be made.

If DBS is acutely and significantly effective in patients who are randomized to receive DBS of the VS/ALIC first, then there is a chance that patients may notice substantial worsening of the pain (assuming that the intervention is effective) and the validity of the experimental blinding may be compromised. In this case, to investigate the effect on blinding, we will record the patient’s and the blinded investigator’s subjective perception (to the best of their ability to guess) of whether active DBS or sham was being delivered during the blinded phase. If the relapse in pain is considered to be threatening or if there is suicidal ideation, then stimulation will be re-initiated and/or patient will be allowed to escape randomization [20,36]. On the other hand, if patients receive sham stimulation first, a relapse in pain will be managed with medical management at that time, because we do not anticipate much change in their baseline pain.

#### Sample size estimation and efficacy

Since the study is designed as a randomized, sham-controlled, double-blinded study, we will also be able to evaluate whether any potential trend indicates efficacy, which would help compute effect sizes and variance in DBS versus sham phases to estimate sample sizes for adequate power in future. Our within-subject, crossover design would also yield the magnitude of placebo effect related to the surgical process itself that is an important practical issue to consider in effect-size estimates for the future.

This study was initially planned to involve a total of 34 patients. The sample size was estimated based on changes in PDI in patients with central pain syndrome in an earlier study [37]. It was determined that to detect an effect size of 0.5 (equivalent to a 7-point decrease in PDI), a total of 34 patients would need to have been included for statistical power of 80% on a two-sided test with a type I error probability of 0.05. The Food and Drug Administration of the United States has, however, determined in its review that the study should initially enroll 10 patients as part of a phase I, safety study.

Due to the small sample size of the proposed pilot phase I trial, procedures for corrections for multiple comparisons cannot be instated; the scope of the subsequent phase II trial would be better suited to include such procedures. By examining only a priori hypotheses, we aim to mitigate the probability of making type I errors. Therefore, we will compare the differences in the primary and secondary outcomes in the third month of DBS with those in the third month of sham stimulation using the paired *t*-test at α = 0.05. Despite the sample size limitation, we expect the study to be informative in estimating the magnitude of the placebo effect after DBS for pain, as well as variances in outcome that to date are unknown. These will be valuable in designing subsequent phase II and III studies.

## Discussion and conclusions

Our protocol aims to test the safety, feasibility and preliminary efficacy of a novel, neuromodulation therapy for intractable TPS. Our approach is based on the premise that failure of traditional cerebral neuromodulation in TPS with DBS [4,5,13,16] emerges from over-reliance on modifying the percept of pain by stimulating sensory-discriminative substrates that may be unviable owing to the offending lesions. Further, since the affective component of pain experience is equally, if not more important than the perceived intensity of pain, targeting regions such as VS/ALIC may successfully modify pain experience without necessarily modifying perception of pain intensity. The level of evidence, however, supporting efficacy for DBS in chronic neuropathic pain is mainly considered class III (descriptive case series, unblinded designs) [38], attributed to poor control of confounders of therapeutic benefit of neuromodulation. While addressing our research question, we have built study design refinements to potentially inform current and future clinical DBS trials in the field.

The ethical-scientific dilemma of introducing a control arm is particularly intense in neurosurgery, given the risk of interventions to the brain. However, since DBS allows for all patients to be implanted at first and then be randomized to active stimulation or sham, studies have explored randomized controlled designs in the use of DBS, particularly in movement disorders [39-42]. Thus, to balance ethical issues (potentially effective treatment compared against control or less effective treatment) with the importance of generating rigorous empirical evidence, we have incorporated a randomized, controlled, crossover design, where both active DBS and sham groups switch to the other intervention after 3 months from their initial randomization. That therapeutic benefits may carry over from the DBS into the sham phase cannot be completely discounted. By evaluating outcomes between active DBS and sham in the third month of corresponding treatments, we expect to minimize the influence of carry over; by noting difference in outcomes between the third month of the initial treatment and the first month of the intervention after crossover, we may also be able to define an adequate time lapse for future crossover studies.

Despite the inclusion of a placebo-controlled phase, altogether, patient expectations from undergoing a clinical-like investigational DBS procedure, therapeutic confusion within the investigative team, and unintentional cues during programming, can introduce several unwanted influences in clinical trial design. By introducing blinding for patients and employing a team where the investigator collecting outcome data is blinded and the surgeon and programming physicians are unaware of outcomes during the blinded phase, we aim to minimize unintentional cues and ensure investigator equipoise [22]. We are also minimizing unintentional cues by allowing adequate time (approximately 1 month) to elapse between programming for DBS and randomization to the intervention group, so that memory of the experience of stimulation is reduced. Nevertheless, a caveat exists. The therapeutic effects may influence patients’ perceptions, and thus, the integrity of experimental blinding [42]. For instance, early effects on mood and anxiety may inflate perceived benefits. To study this possibility, we will document the frequency with which patients can guess the allocated treatment and we will relate the Beck depression and anxiety inventories with PDI to note whether the placebo of DBS confounds therapeutic differences from the sham. We acknowledge, however, that small sample size may limit our ability to draw concrete inferences.

Finally, to allow systematic study of efficacy, adequate controls in enrollment and analysis would be critical. We are maintaining diagnostic consistency by restricting inclusion only to TPS etiology. Although such a step limits the sample size, it addresses the criticism of previous DBS studies where heterogeneity of etiologies and targets affected concrete conclusions about therapeutic efficacy [8]. With regards to analysis, instead of using the typical 50% response on the VAS as the success criterion, we base our choice of outcomes upon the intended therapeutic benefit. Because the affective experience of pain, rather than pain intensity itself, may be influenced with DBS of VS/ALIC, the Pain Disability Index has been chosen instead, and a success criterion with this measure has been defined at 40%. Since we anticipate a relation between affective and cognitive benefits and alleviation of pain disability, detailed neuropsychological pain, and cognitive and psychiatric assessments are explored to study interactions between pain, mood, anxiety, and depression, as well as personality and memory/experience, and the overall effect on quality of life. Long-term follow-up time points have been built into the design to allow calculation of long-term efficacy or stimulation tolerance. Last, our pilot study also represents the first attempt at investigating the proof-of-mechanism of a novel target of neuromodulation in TPS so that the influence of such targeting on pain networks can be described.

The benefits of DBS in chronic neuropathic pain, especially in TPS, are still less than adequate, to justify approval for widespread clinically labeled use. We propose that identifying better clinical alternatives in DBS may potentially improve efficacy of the approach. More importantly, however, it is incumbent on the field to incorporate well-controlled, blinded trial designs to explore the benefits of current as well as novel DBS targets. Based on recent recommendations, our trial involving DBS of the VS/ALIC may serve as an example of feasibly creating protocols that improve the quality of the evidence base.

### Trial status

The trial is ongoing and is actively enrolling.

## Abbreviations

ALIC: anterior limb of internal capsule; BDI: Beck Depression Inventory; C-SSRS: Columbia-Suicide Severity Rating Scale; DBS: deep brain stimulation; DSMB: Data Safety and Monitoring Board; EuroQOL(EQ-5D): Euro Quality of Life scale; fMRI: functional magnetic resonance imaging; IPG: implantable pulse generator; MADRS: Montgomery-Asburg Depression Rating Scale; MCS: motor cortex stimulation; MEG: Magneto-encephalography; MRI: magnetic resonance imaging; NEO_FFI: NEO Five Factor Inventory; PANAS: Positive and Negative Affect Schedule; PDI: Pain Disability Index; TPS: thalamic pain syndrome; VAS: visual analog scale; VS: ventral striatum.

## Competing interests

AM has the following conflicts of interest to disclose, none of which are directly pertinent to this research: Intellect Medical (advisory board, consultant, share holder), ATI and Cardionomics (shareholder), and Monteris (consultant).

## Authors’ contributions

All authors made significant intellectual contributions. AM is the principal investigator. AM and DM developed the approach discussed here as an intervention. EP created the first draft, which was reviewed and edited critically by all other authors, and was included in revisions by EP. All authors read and approved the final manuscript.

## Authors’ information

EP is project staff in Departments of Biomedical Engineering, Physical Medicine and Rehabilitation and Center for Neurological Restoration at the Cleveland Clinic.

DM is the chairman of Department of Psychiatry & Psychology and Center for Behavioral Health at the Cleveland Clinic.

AM is the Director of the Center for Neurological Restoration, Department of Neurological Surgery at the Cleveland Clinic.
